# Disseminated nontuberculous mycobacteria infection in an immunocompetent host: A case report

**DOI:** 10.1097/MD.0000000000032416

**Published:** 2023-01-06

**Authors:** Hye Soon Shin, Bumhee Yang, So Rae Kim, Hee-Sung Kim, Kyeong Seob Shin, Yoon Mi Shin

**Affiliations:** a Department of Internal Medicine, Chungbuk National University Hospital, Chungbuk National University College of Medicine, Cheongju, Korea; b Division of Pulmonary and Critical Care Medicine, Department of Internal Medicine, Chungbuk National University Hospital, Chungbuk National University College of Medicine, Cheongju, Korea; c Division of Infectious Disease, Department of Internal Medicine, Chungbuk National University Hospital, Chungbuk National University College of Medicine, Cheongju, Korea; d Department of Laboratory Medicine, Chungbuk National University Hospital, Chungbuk National University College of Medicine, Cheongju, Korea.

**Keywords:** catheter-related bloodstream infection, disseminated nontuberculous mycobacterium infection, immunocompetent, Mycobacterium, *Mycobacterium massiliense*, rapidly growing

## Abstract

Disseminated nontuberculous mycobacterial (NTM) infections are rare and occur primarily in immunocompromised hosts. *Mycobacterium abscessus complex* (MABC), including *M abscessus subsp. massiliense* (hereafter *M massiliense*) is a complex of rapidly growing mycobacterial (RGM) species of NTM. Here, we present a rare case of disseminated NTM infection with RGM bacteremia caused by *M massiliense* in an immunocompetent host.

**Patient concerns:**

A 64-year-old woman with a recent history of spine fracture and septic pneumonia was transferred to our emergency room for dyspnea and fever. A peripherally inserted central catheter (PICC) had been placed over 6 months prior.

**Diagnoses:**

Chest computed tomography (CT) showed multifocal patchy consolidations and ground-glass opacity in both lungs. NTM suspected of RGM was isolated from the blood cultures. During hospitalization, multiple erythematous and hemorrhagic crusted nodules developed on the patient’s upper and lower extremities, which were confirmed as disseminated NTM infection on skin biopsy.

**Interventions:**

After NTM suspected of RGM was isolated from the blood cultures, the patient was empirically treated with antibiotics used for NTM infection, and the PICC was removed. Thereafter, the subspecies of NTM was reported as *M massiliense* and she was treated according to the antibiotic susceptibility testing results.

**Outcome:**

Although skin lesions and inflammatory markers improved gradually during antibiotic treatment over 10 weeks, NTM could still be isolated from the blood culture.

**Lessons:**

Disseminated NTM infections with RGM bacteremia in an immunocompetent host have rarely been reported. In this case, PICC placement for more than 6 months was suspected to be an important risk factor for RGM bacteremia in an immunocompetent patient. To date, there are only insufficient case reports, moreover no clear guidelines regarding the optimal choice of antibiotics or length of treatment for disseminated NTM infection. Therefore, it is necessary to establish treatment guidelines for patients with disseminated NTM infection and bacteremia.

## 1. Introduction

The term, “Nontuberculous mycobacteria (NTM)” generally refers to mycobacteria other than the *Mycobacterium tuberculosis complex* and *Mycobacterium leprae*.^[1]^ In Korea, the frequency of detection of NTM was reported to be in the order of *Mycobacterium avium complex* (MAC), *Mycobacterium abscessus complex* (MABC), and *Mycobacterium kansasii*.^[2]^ MABC is a group of rapidly growing mycobacteria (RGM) and is classified into 3 subspecies: *M abscessus subsp. abscessus* (hereafter *M abscessus), M abscessus subsp. bolletii* (hereafter *M bolletii), and M abscessus subsp. massiliense* (hereafter *M massiliense*).^[3,4]^

Human diseases caused by NTM are classified into 4 distinct clinical syndromes: chronic pulmonary disease, lymphadenitis, cutaneous disease, and disseminated disease.^[5]^ Disseminated infections are known to occur in immunocompromised hosts, such as those with AIDS, hematologic malignancy, or a history of immunosuppressive therapy.^[6]^ In addition, although the number of RGM infections is increasing in Korea,^[2]^ reports of disseminated RGM infections, including bacteremia, are still rare.

In this report, we present a rare case of an immunocompetent patient with disseminated NTM infection with bacteremia due to *M massiliense*.

## 2. Case presentation

**Figure 1.**
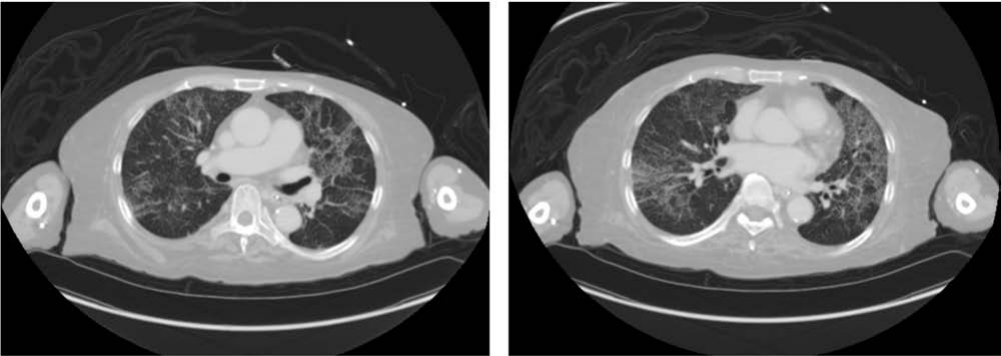
Initial chest X-ray and computed tomographic findings. multifocal patchy consolidations and ground-glass opacity (GGO) in both lungs.

A 64-year-old woman was transferred to the emergency room for dyspnea and fever. She had recently been hospitalized for a spinal fracture and septic pneumonia in a local hospital and was transferred with a peripherally inserted central catheter (PICC). When she visited our center, the PICC had been in place for more than 6 months. On arrival, her vital signs were as follows: blood pressure, 150/100 mm Hg; heart rate, 150 beats/min (irregular); respiratory rate, 30 breaths/min; oxygen (O_2_) saturation 88% (on room air); body temperature 38.0°C. Initial laboratory results showed a white blood cell count of 10,920/µL, with 86.3% neutrophils and C-reactive protein (CRP) 9.02 mg/dL. Chest computed tomography (CT) showed multifocal patchy consolidations and ground-glass opacity in both lungs (Fig. 1).

Blood (both from peripheral and central line), sputum and urine cultures were performed during the emergency room stay, and she was admitted to the intensive care unit (ICU). Empirical treatment for pneumonia consisting of intravenous (IV) piperacillin/tazobactam (4.5 g every 6 hours) and levofloxacin (500 mg/day) was administered. After admission to the ICU, high-flow nasal cannula oxygen therapy was administered for persistent respiratory distress; however, the O_2_ saturation was less than 80%, and the patient was intubated and started on mechanical ventilation.

On hospital day 5, while waiting for culture results, the patient developed fever and elevated CRP levels; therefore, antibiotic treatment was changed to IV teicoplanin and meropenem. Subsequently, the patient showed improvement in fever and inflammatory markers, including white blood cell count and CRP.

**Figure 2.**
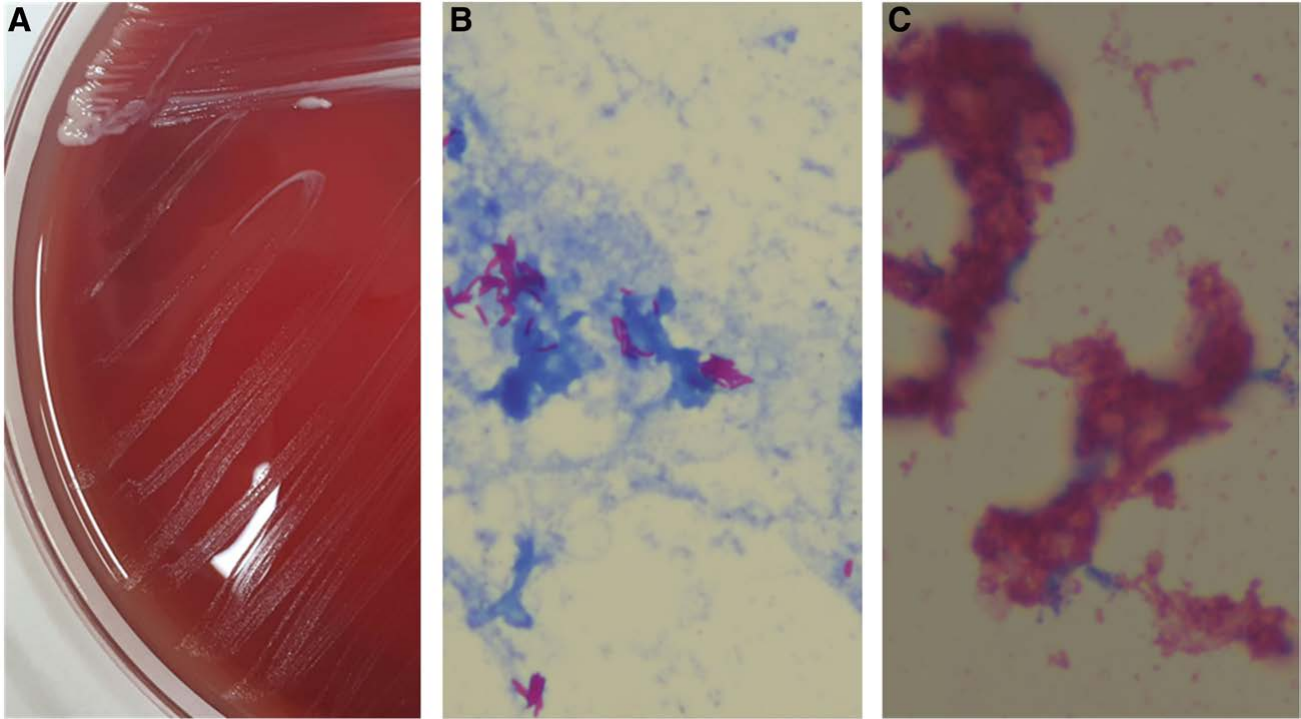
A positive blood culture with rapidly growing mycobacteria. (A) Colony finding of *Mycobacterium abscessus*. Small and gray colony were rapidly grown after 3-day incubation in blood agar plate. (B) Gram stain finding of blood culture (×1000). Gram positive filamentous bacilli with less staining are frequently noted from blood culture bottle with growing signal. (C) Acid fast stain of blood culture (Ziehl-Neelsen staining, ×1000). Acid fast bacilli (red color) are frequently noted in blood from culture bottle with growing signal.

On hospital day 7, The preliminary report of blood cultures was suspected of NTM, especially RGM (Fig. 2). After isolation of the NTM, teicoplanin and meropenem were stopped, and the PICC was removed. In addition, the patient was administered IV imipenem/cilastatin 500 mg every 8 hours, amikacin 15 mg/kg/day, and azithromycin 500 mg.

**Figure 3.**
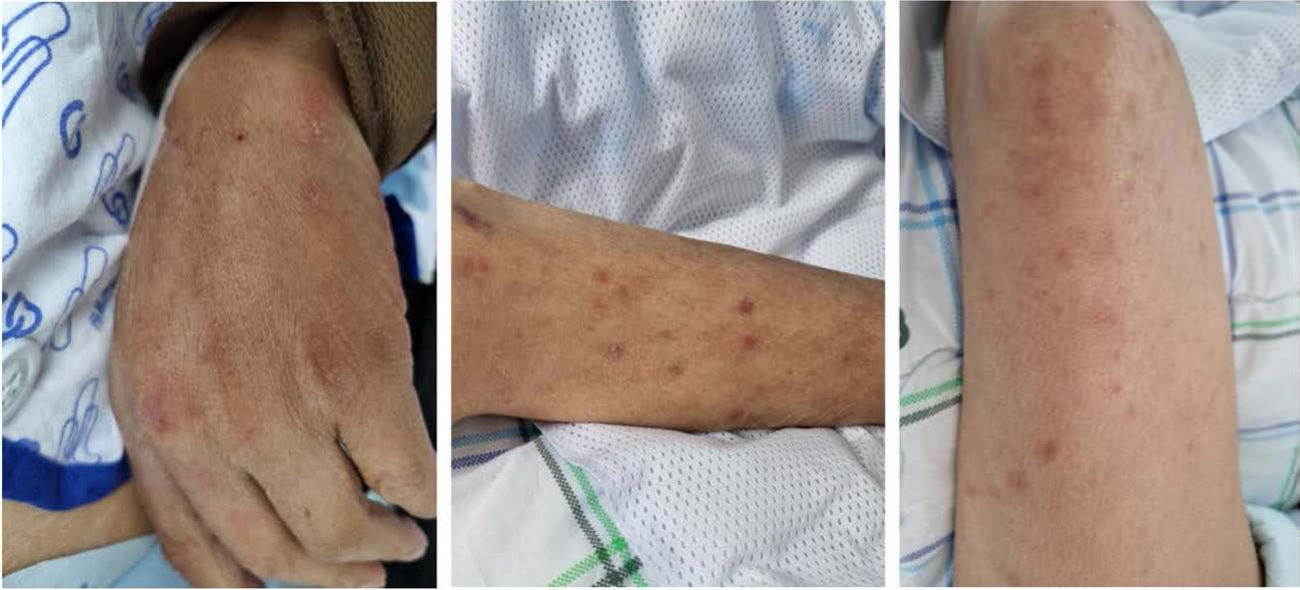
Multiple erythematous nodules and hemorrhagic crusted nodules on the both upper and lower extremities.

On hospital day 9, the patient’s clinical symptoms, chest imaging, and inflammatory markers were all improved, the patient was extubated and O_2_ saturation was maintained stably via a nasal cannula. The patient was transferred to the general ward. During the ICU stay, multiple erythematous and hemorrhagic crusted nodules developed on the patient’s upper and lower extremities (Fig. 3). On hospital day 12, the patient underwent skin biopsy to confirm disseminated NTM infection. The biopsy result showed chronic granulomatous inflammation with acid-fast bacilli, consistent with mycobacterium infection. After the diagnosis of disseminated NTM infection with bacteremia, the patient underwent laboratory tests to confirm humoral immunity. The T lymphocyte subset counts for CD3, CD4, and CD8 were normal.

On hospital day 18, the result of NTM polymerase chain reaction confirmed the presence of NTM subspecies and the patient was diagnosed with *M massiliense*. On hospital day 31, the antibiotic susceptibility testing was reported (Table 1). Based on these results, the patient was maintained on 4 medications; amikacin, imipenem, azithromycin, tigecycline, and linezolid.

**Table 1:** Susceptibility testing for the *Mycobacterium massiliense* isolate.

Antibiotics	MIC (mcg/mL)	Interpretation
Clarithromycin	≤0.5	S
Amikacin	8	S
Moxifloxacin	>8	R
Linezolid	8	S
Streptomycin	>64	NI
Ciprofloxacin	8	R
Doxycycline	>16	R
Clofazimine	0.5	NI
Trimethoprim/Sulfamethoxazole	8/152	R
Cefoxitin	32	I
Imipenem	4	S
Meropenem	16	I
Tobramycin	8	R
Tigecycline	1	NI

I = intermediate, MIC = minimum inhibitory concentration, NI = no Clinical and Laboratory Standards Institute (CLSI) interpretive guidelines for this antibiotic, R = resistant, S = susceptible.

During antibiotic treatment over 10 weeks, the skin lesions gradually improved. However, NTM was still identified in the blood culture. Nevertheless, at the patient’s strong request, she was discharged with 2 sensitive oral drugs (azithromycin and linezolid). However, 10 days after discharge, the patient eventually died due to a sudden deterioration (Supplemental figure 1, http://links.lww.com/MD/I185).

## 3. Discussion

Here we present a rare case of disseminated NTM infection in an immunocompetent host with RGM bacteremia caused by *M massiliense*. Considering disseminated NTM infections and RGM bacteremia occur primarily in immunocompromised hosts, our case is interesting because it developed in a patient with normal immunity.

Most disseminated NTM infections, particularly blood stream infection, have been reported in HIV-infected or immunocompromised individuals. Although there are not enough cases of disseminated NTM infections in immunocompetent patients (supplemental table 1, http://links.lww.com/MD/I186), they are usually not as fatal as other bacterial bloodstream infections. It is known that the best chance of cure (more than 90%) is obtained with a combination of at least 2 active antimicrobials given for a minimum of 4 weeks, plus removal of the intravascular catheter.^[7]^ In previous limited datas, disseminated infection (≥ 2 sites of infection) is considered a factor associated with poor outcome. In a previous case report of NTM bacteremia in an immunocompetent patient, NTM was negative in blood after 3 days of antibiotic therapy; in contrast, in our case, disseminated infection including blood and skin was persistent after 10 weeks of antibiotic therapy. More cases are needed to compare the prognosis of disseminated NTM infections in immunocompetent hosts.

Catheter-related infections are associated with RGM bacteremia.^[7]^ RGMs can form biofilms and colonize in catheters.^[8,9]^ Several studies^[8–10]^ have established that almost all species of RGM can form biofilms, which enhance their ability to cause catheter-related bloodstream infections. MABC causes faster biofilm growth than other RGM, which is believed to contribute to the pathogenicity of this more virulent organism.^[11]^ In our case, the patient was transferred from a local hospital with a PICC for more than 6 months. As such, the central venous catheter is considered an important risk factor for RGM bacteremia in immunocompetent hosts.

It is important to note for our case that there are only scanty treatment guidelines outlining the optimal choice of antibiotics or length of treatment necessary for the management of NTM bloodstream infections, and therefore, each treatment depends on the expert opinion. A major challenge in managing MACB is that the pathogens are highly drug resistant. The guideline from the American Thoracic Society/Infectious Diseases Society of America^[1]^ recommends macrolide-based multidrug therapy including IV amikacin with imipenem (or cefoxitin), based on the results of drug susceptibility testing. However, the outcomes are unsatisfactory, and long-term use of injectable antibiotics is not feasible.^[12,13]^ We administered recommended regimen for this patient including amikacin with imipenem azithromycin and linezolid for more than 10 weeks according to the drug susceptibility testing results, and although her clinical symptoms and skin lesions were improved, the blood culture results showed persistent positivity. At the patient’s strong request, she was discharged with 2 sensitive oral medications, but eventually died due to a sudden deterioration. Therefore, we believe that long-term antibiotic therapy including IV drugs will be necessary until the blood culture is negative for disseminated NTM infection accompanied by bacteremia and it is necessary to accumulate more treatment guidelines for patients with disseminated infection.

In conclusion, we report a rare case of disseminated NTM infection with *M massiliense* bacteremia in an immunocompetent host. The ideal treatment regimen for disseminated MABC infections accompanying bacteremia should be determined based on precise species identification and antimicrobial susceptibility testing; however, data on the treatment duration are still lacking. Therefore, the accumulation of more cases is necessary to determine the optimal management of disseminated MABC infections with bacteremia.

## Author contributions

**Conceptualization:** Bumhee Yang, Kyeong Seob Shin, Yoon Mi Shin.

**Data curation:** Hye Soon Shin, Bumhee Yang, Kyeong Seob Shin, Yoon Mi Shin.

**Formal analysis:** Hye Soon Shin, Bumhee Yang, So Rae Kim, Yoon Mi Shin.

**Investigation:** Hee-Sung Kim, Kyeong Seob Shin.

**Methodology:** Kyeong Seob Shin.

**Project administration:** Yoon Mi Shin.

**Resources:** Kyeong Seob Shin, Yoon Mi Shin.

**Supervision:** Yoon Mi Shin.

**Validation:** Yoon Mi Shin.

**Writing – original draft:** Hye Soon Shin, Bumhee Yang.

**Writing – review & editing:** Bumhee Yang, So Rae Kim, Hee-Sung Kim, Kyeong Seob Shin, Yoon Mi Shin.

## Supplementary Material



## References

[1] Koh WJ. Nontuberculous mycobacteria-overview. Microbiol Spectr. 2017;5:10.1128/microbiolspec.TNMI7-0024-2016. 10.1128/microbiolspec.tnmi7-0024-2016 PMC11687458 28128073

[2] Lee H Myung W Koh WJ. Epidemiology of nontuberculous mycobacterial infection, South Korea, 2007-2016. Emerg Infect Dis. 2019;25:569–72. 30789139 10.3201/eid2503.181597 PMC6390769

[3] Bryant JM Grogono DM Greaves D. Whole-genome sequencing to identify transmission of *Mycobacterium abscessus* between patients with cystic fibrosis: a retrospective cohort study. Lancet. 2013;381:1551–60. 10.1016/S0140-6736(13)60632-7 PMC3664974 23541540

[4] Kumar K Daley CL Griffith DE. Management of Mycobacterium avium complex and *Mycobacterium abscessus* pulmonary disease: therapeutic advances and emerging treatments. Eur Respir Rev. 2022;31:210212. 10.1183/16000617.0212-2021 PMC9488909 35140106

[5] Griffith DE Aksamit T Brown-Elliott BA. An official ATS/IDSA statement: diagnosis, treatment, and prevention of nontuberculous mycobacterial diseases. Am J Respir Crit Care Med. 2007;175:367–416. 17277290 10.1164/rccm.200604-571ST

[6] Horsburgh CR Jr. Mycobacterium avium complex infection in the acquired immunodeficiency syndrome. N Engl J Med. 1991;324:1332–8. 2017230 10.1056/NEJM199105093241906

[7] El Helou G Viola GM Hachem R. Rapidly growing mycobacterial bloodstream infections. Lancet Infect Dis. 2013;13:166–74. 23347634 10.1016/S1473-3099(12)70316-X

[8] Hawkins C Qi C Warren J. Catheter-related bloodstream infections caused by rapidly growing nontuberculous mycobacteria: a case series including rare species. Diagn Microbiol Infect Dis. 2008;61:187–91. 18294801 10.1016/j.diagmicrobio.2008.01.004

[9] Raad II Vartivarian S Khan A. Catheter-related infections caused by the Mycobacterium fortuitum complex: 15 cases and review. Rev Infect Dis, 1991;13:1120–5. 1775845 10.1093/clinids/13.6.1120

[10] Lee SA Raad II Adachi JA. Catheter-related bloodstream infection caused by Mycobacterium brumae. J Clin Microbiol. 2004;42:5429–31. 15528764 10.1128/JCM.42.11.5429-5431.2004 PMC525179

[11] Esteban J Martín-de-Hijas NZ Fernandez AI. Epidemiology of infections due to nonpigmented rapidly growing mycobacteria diagnosed in an urban area. Eur J Clin Microbiol Infect Dis. 2008;27:951–7. 18458972 10.1007/s10096-008-0521-7

[12] Koh WJ Jeon K Lee NY. Clinical significance of differentiation of Mycobacterium massiliense from *Mycobacterium abscessus*. Am J Respir Crit Care Med. 2011;183:405–10. 20833823 10.1164/rccm.201003-0395OC

[13] Park J Cho J Lee CH. Progression and treatment outcomes of lung disease caused by *Mycobacterium abscessus* and *Mycobacterium massiliense*. Clin Infect Dis. 2017;64:301–8. 10.1093/cid/ciw723 28011609

