# Supplementary figures and images for: Disseminated nontuberculous mycobacteria infection in an immunocompetent host: A case report
Source: Medicine (Baltimore). 2023 Jan 6;102(1):e32416. doi: 10.1097/MD.0000000000032416 (PMC9829286; doi:10.1097/MD.0000000000032416)

## Slide 1
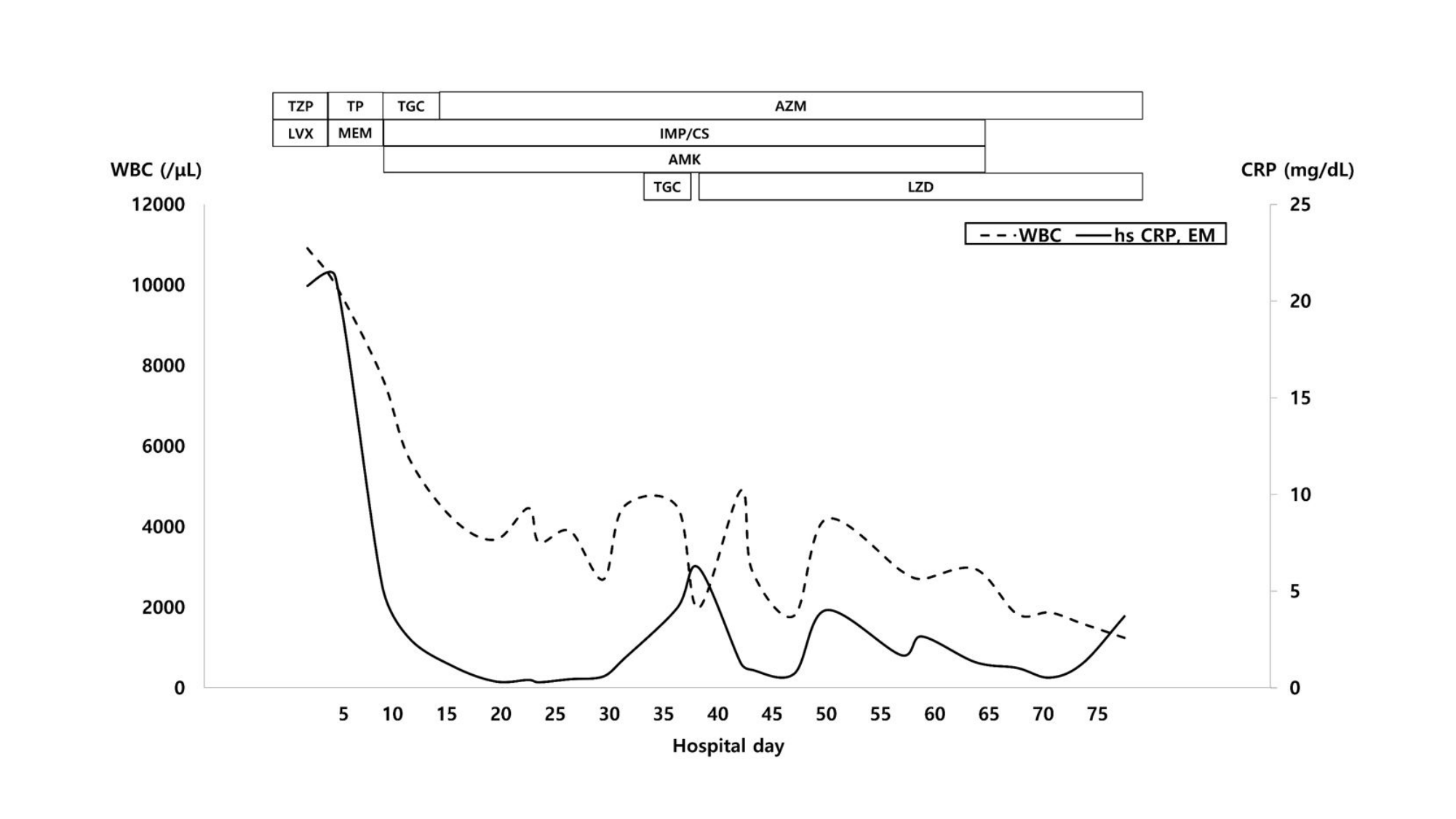

Supplement: Supplementary file 1 [file medi-102-e32416-s001.pptx]
