# Supplementary material for: Disseminated nontuberculous mycobacteria infection in an immunocompetent host: A case report
Source: Medicine (Baltimore). 2023 Jan 6;102(1):e32416. doi: 10.1097/MD.0000000000032416 (PMC9829286; doi:10.1097/MD.0000000000032416)
Supplement: Supplementary file 2 [file medi-102-e32416-s002.pdf]

Supplemental table 1. Reported cases of disseminated nontuberculous mycobacteria infection

Author, year [Ref]	No. of patients	Organism (no.)	Site (no.)	Immunocompromised host	Antibiotics/Duration (weeks)	Outcome
Oka K, 2021[1]	1	*Mycobacterium abscessus subsp. massiliense*	Skin, Joint	Yes	imipenem/cilastatin, amikacin, clarithromycin, sitafloxacin	Died
de Melo Carvalho R, 2020[2]	1	*Mycobacterium chimaera*	Blood, Bone marrow, Lungs	Yes	clarithromycin, rifabutin, ethambutol, amikacin, linezolid, moxifloxacin / 12 months	cultures were negative after 4 months of therapy
N. Beydoun, 2020[3]	1	*Mycobacterium mucogenicum*	Blood	No	meropenem, azithromycin, ciprofloxacin / 4 weeks	cultures were negative after 3 days of therapy
P Chetchotisakd, 2007[4]	129	*Mycobacterium abscessus (45)* *Mycobacterium fortuitum (11)* *Mycobacterium chelonae (9)* *Mycobacterium*	Lymph node (115) Skin and soft tissue (34) Lung (25) Bone and joint (23)	Not specified	Not specified	Not specified

		thermoresistibile (1)<br>*Mycobacterium avium complex (9)*<br>*Mycobacterium simiae (2)*<br>*Mycobacterium scrofulaceum (2)*<br>*Mycobacterium szulgai (2)*<br>*Mycobacterium kansasi (1)*<br>*Mycobacterium malmoense (1)*<br>*Mycobacterium haemophilum (1)*<br>*other species (9)*<br>*not identified (41)*	Blood (21)<br>Liver (19)<br>Spleen (12)<br>Sinus and tonsil (10)<br>CNS (9)<br>Breast (3)<br>Peritoneum (1)<br>Pleura (1)<br>Tongue (1)<br>Small bowel (1)<br>Bone marrow (1)			
Saira Butt, 2019[5]	1	*Mycobacterium smegmatis (1)*	Blood	No	meropenem, amikacin, ethambutol, doxycycline / 8	clinically stable

Masako Mizusawa [6]	17	*Mycobacterium fortuitum (9) Mycobacterium. abscessus, chelonae (1) rapidly growing mycobacterium (7)*	blood	No	Ceftriaxone, Vancomycin, Nafcillin, Cefepime, Daptomycin, Piperacillin/ Tazobactam, Gentamicin. / Not specified duration	Not specified

[1] Oka K, Morioka H, et al. Bursitis, Bacteremia, and Disseminated Infection of Mycobacteroides (Mycobacterium) abscessus subsp. Massiliense. The Japanese Society of Internal Medicine. Intern Med 2021 Sep 15;60(18):3041-3045. doi: 10.2169/internalmedicine.6189-20. Epub 2021 Mar 29.

[2] de Melo Carvalho R, Nunes AL, Sa R, Ramos I, Valente C, Saraiva da Cunha J. Mycobacterium chimaera disseminated infection. J Med Cases. 2020 Feb;11(2):35-36. doi: 10.14740/jmc3420. Epub 2020 Feb 28

[3] N. Beydoun, Z. Wiley, N. Rouphael. Mycobacterium mucogenicum bacteremia in an immunocompetent host: A case report and concise review. IDCases 2021;23:e01032. doi: 10.1016/j.idcr.2020.e01032.

[4] Chetchotisakd P, Kiertiburanakul S, Mootsikapun P, et al. Disseminated nontuberculous mycobacterial infection in patients who are not infected with HIV in Thailand. Clin Infect Dis 2007 Aug 15;45(4):421-7. doi: 10.1086/520030. Epub 2007 Jul 5.

[5]Butt S, Tirmizi A. Mycobacterium smegmatis bacteremia in an immunocompetent host. IDCases 2019 Mar 12;15:e00523. doi: 10.1016/j.idcr.2019.e00523.

[6] Mizusawa M, Vindenes T, Buckley S, Armstrong. A case series of rapidly growing mycobacterial catheter-related bloodstream infections among immunocompetent patients. J Clin Tuberc Other Mycobact 2020 Oct 19;21:100196. doi: 10.1016/j.jctube.2020.100196. eCollection 2020 Dec.
